# Loss of PICH promotes chromosome instability and cell death in triple-negative breast cancer

**DOI:** 10.1038/s41419-019-1662-6

**Published:** 2019-06-03

**Authors:** Yan Huang, Wanjin Li, Weiwei Yan, Jiaqi Wu, Liang Chen, Xiaohong Yao, Feng Gu, Luye Lv, Jiangman Zhao, Ming Zhao, Tian Xia, Qiuying Han, Teng Li, Xiaomin Ying, Tao Li, Qing Xia, Ailing Li, Xuemin Zhang, Yuan Chen, Tao Zhou

**Affiliations:** 1 State Key Laboratory of Proteomics, Institute of Basic Medical Sciences, National Center of Biomedical Analysis, Beijing, 100850 China; 2 Chinese PLA General Hospital, Beijing, 100853 China; 3 Center of Computational Biology, Beijing Institute of Basic Medical Sciences, Beijing, 100850 China; 4 Institute of Pathology and Southwest Cancer Center, Southwest Hospital, Third Military Medical University (Army Medical University), Chongqing, 400038 China; 5 Department of Breast Pathology, Tianjin Medical University Cancer Institute and Hospital, Tianjin, 300060 China; 6 Research Institute of Jilin University, the First Hospital of Jilin University, Changchun, Jilin Province 130021 China

**Keywords:** Breast cancer, Apoptosis

## Abstract

Triple-negative breast cancer (TNBC), defined by the lack of expression of estrogen, progesterone, and ERBB2 receptors, has the worst prognosis of all breast cancers. It is difficult to treat owing to a lack of effective molecular targets. Here, we report that the growth of TNBC cells is exceptionally dependent on PICH, a DNA-dependent ATPase. Clinical samples analysis showed that PICH is highly expressed in TNBC compared to other breast cancer subtypes. Importantly, its high expression correlates with higher risk of distal metastasis and worse clinical outcomes. Further analysis revealed that *PICH* depletion selectively impairs the proliferation of TNBC cells, but not that of luminal breast cancer cells, in vitro and in vivo. In addition, knockdown of *PICH* in TNBC cells induces the formation of chromatin bridges and lagging chromosomes in anaphase, frequently resulting in micronucleation or binucleation, finally leading to mitotic catastrophe and apoptosis. Collectively, our findings show the dependency of TNBC cells on PICH for faithful chromosome segregation and the clinical potential of *PICH* inhibition to improve treatment of patients with high-risk TNBC.

## Introduction

Breast cancer is a heterogeneous disease that includes several subtypes with remarkably different biological features and clinical behavior^1–4^. Hormone (estrogen or progesterone) receptor-positive breast cancer and ERBB2 (HER2)-positive breast cancer currently account for 75–80% and 15–20% of breast cancer cases, respectively, with about half of ERBB2-positive cases co-expressing hormone receptors.The remaining 10–15% of breast cancers are defined as triple-negative breast cancers (TNBC), and they are characterized by the lack of ER, PR, and HER2 expression^5,6^. Unlike tumors that are hormone receptor- and/or HER2-positive, triple-negative tumors lack established therapeutic targets. As a result, conventional chemotherapy is the only effective systemic treatment for this disease. Patients with TNBC have a higher rate of distant recurrence and a poorer prognosis than patients with other breast cancer subtypes^7–9^. TNBC tumors grow rapidly, and are generally larger in size and of higher grade. Moreover, TNBC have lymph node involvement at diagnosis, and are biologically more aggressive^10–12^. Insights into the processes that regulate TNBC growth and metastases, as well as the drivers of these processes, could contribute to discovering novel therapeutic targets and approaches for high-risk patients.

Sporadic TNBC shares many phenotypical and molecular features with BRCA1-associated cancers^13–15^. BRCA1 plays an important role in DNA double-strand break repair, contributing to the maintenance of DNA stability^16^. Therefore, defects in the DNA repair pathway could provide a promising therapeutic target for this subgroup of patients with TNBC. Poly(ADP-ribose) polymerase (PARP) inhibitors exploit this deficiency through synthetic lethality and have emerged as promising targeted therapies^17–19^. PARP inhibition compromises replication fork stability and leads to DNA lesions. During mitosis, these DNA lesions can cause chromatin bridges and lead to cytokinesis failure, multinucleation, and cell death^20^. Compared with the luminal subtype of breast cancer, TNBC have more frequent *TP53* gene mutations and epidermal growth factor receptor (EGFR) expression, display exceedingly high levels of proliferation-related genes and high mitotic index^4,10,13^. Paclitaxel is an effective and widely used anti-mitotic drug that targets microtubule in TNBC. Low concentrations of paclitaxel can cause a mitotic block followed by aberrant mitosis, multiple micronuclei, and tetraploidy, which may be followed by apoptosis^21–23^. Both PARP inhibitors and paclitaxel treatment for TNBC cells can induce mitotic catastrophe, characterized by the occurrence of dysregulated mitosis or missegregation of the chromosomes, followed by aberrant cell division. We hypothesized that, due to its intrinsic genetic complexity, TNBC may have a unique cell cycle progression mechanism and be sensitive to drugs that cause mitosis dysregulation.

The Plk1-interacting checkpoint helicase (PICH) was originally identified as a binding partner and substrate of polo-like kinase 1 (Plk1), a major regulator of M phase progression^24^. It translocates from the cytoplasm to the centromere/kinetochore (KT) region of condensed chromosomes at the onset of mitosis^24,25^ and during anaphase, decorates the ultrafine DNA bridges that connect separating chromatids^24,26^. *PICH* deletion in chicken and human cells increases the occurrence of chromosomal abnormalities, such as chromatin bridges, micronuclei, and binucleation^27,28^. *Pich* knockout mice embryos exhibit DNA damage, p53 activation, and apoptosis^29^. *PICH* expression has been reported to be elevated in breast cancer^30^, but its role in TNBC is largely unknown. Here, we analyzed PICH in TNBC clinical samples and in breast cancer cell lines, and investigated whether it plays a role in the growth of TNBC. Analyses of breast cancer patient data according to subtypes revealed a remarkable overexpression of PICH in TNBC. We discovered that PICH is essential in triple-negative, but not in luminal breast cancer cells, in vitro and in vivo. Notably, high expression of PICH promoted the growth of TNBC cells and ensured faithful chromosome segregation. Together, our data demonstrate that PICH is a novel TNBC prognostic marker, and indicate the importance of further investigation of PICH in TNBC-targeted therapies.

## Results

### PICH expression is elevated in TNBC and associated with poor outcomes

**Fig. 1:**
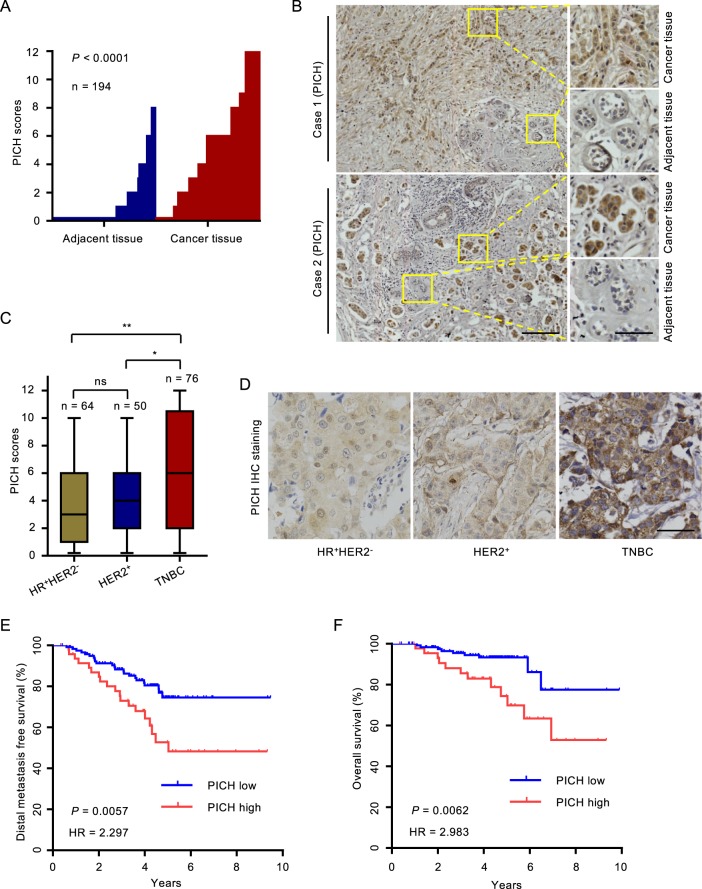
*PICH* expression is elevated in TNBC and associated with poor outcomes. **a** PICH immunohistochemical scores in breast cancer tumors and matched adjacent normal breast epithelium. *P*-value was determined by the Mann–Whitney *U* test; *n* = 194. **b** Representative images from immunohistochemical staining of PICH in tumors from two cases of breast cancer. The boxed areas in the left images are magnified in the right images. Scale bars, 150 µm (left), 50 µm (right). **c** Box plot of PICH expression in breast cancer from patient subjects divided in the HR^+^HER2^−^, HER2^+^, and TNBC subgroups (four patients without complete information on receptor expression were excluded). Differences between groups were analyzed by using a two-tailed unpaired *t*-test. *P < 0.05, **P < 0.01; ns, not significant. **d** Representative images of PICH staining in the three groups described in (**c**). Scale bar, 50 µm. **e**, **f** Kaplan–Meier estimates of distal metastasis-free survival (**e**) and overall survival (**f**) of the patients with breast cancer (*n* = 169, the survival information for 25 of the 194 subjects was unavailable). Comparison was made between the high PICH expression (score ≥ 6) and low PICH expression (score < 6) groups. Marks on graph lines represent censored samples. *P*-value refers to two-sided log-rank test

To investigate the potential clinical role of PICH in breast cancer, we collected breast cancer and the matched adjacent normal tissue samples from 194 human subjects and performed IHC using an anti-PICH antibody, the specificity of which we first confirmed (Supplementary Fig. S1A). The data showed that PICH expression was highly elevated in breast tumor tissues compared with matched adjacent normal tissues (Fig. 1a, b). We then classified breast cancer subjects into three groups according to their ER, PR, and HER2 protein expression status, and found that PICH expression was greatly elevated in TNBC tumors compared with hormone receptor (HR)-positive/HER2-negative tumors, and HER2-positive tumors (Fig. 1c, d). To assess the clinical relevance of PICH expression, we analyzed the survival of breast cancer subjects. As shown in Fig. 1e, f, patients with tumors with highly expressed PICH showed significantly higher risk of distal metastasis and poorer overall survival than those with tumors with low PICH expression. Further, we interrogated three independent published data sets: The Cancer Genome Atlas (TCGA) Breast^31^, the METABRIC^32^ and the GSE12276^33^ cohorts (BCIP; http://www.omicsnet.org/bcancer/) and found that *PICH* mRNA levels were significantly higher in TNBCs than in non-TNBCs (Supplementary Fig. S1B-D). Similarly, we used two additional public independent microarray datasets (GSE25055, GSE11121)^34,35^ and found that increased *PICH* expression was associated with diminished distal metastasis-free survival (Supplementary Fig. S1E-F). These findings indicate that PICH expression is upregulated in TNBC cancer and high levels of PICH expression are associated with poor outcome in the patients.

### Loss of PICH selectively impairs TNBC cell viability and proliferation

**Fig. 2:**
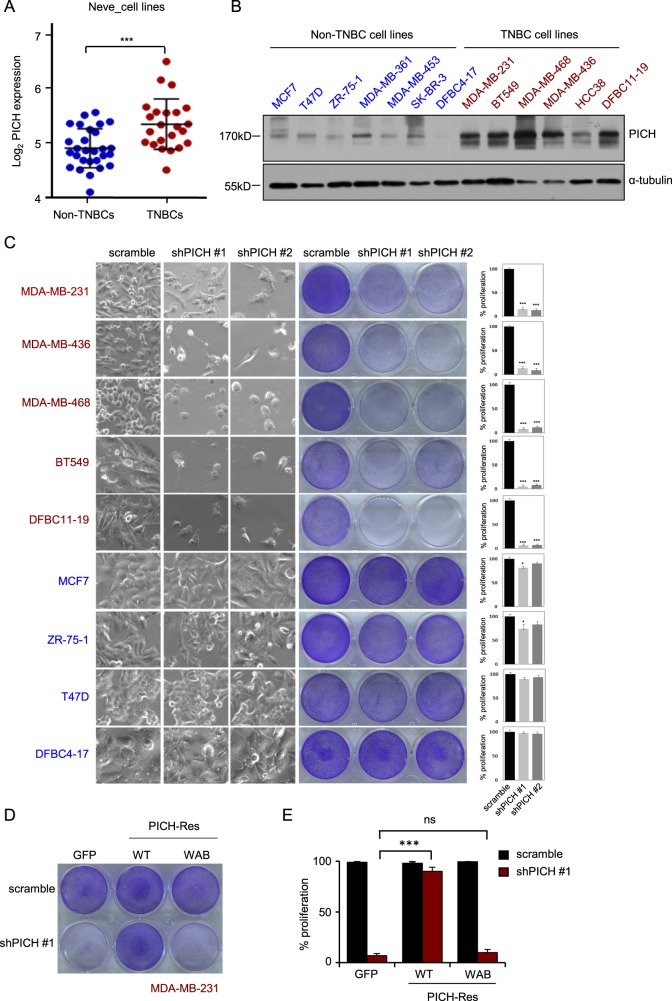
Loss of *PICH* selectively impairs TNBC cell viability and proliferation. **a** The PICH mRNA data in 28 established non-TNBC and 23 TNBC cell lines were obtained from the Neve dataset^34^ and are shown as a dot chart. **b** PICH protein expression, as assessed by western blotting, in a panel of breast cancer cell lines, including seven non-TNBC cell lines and six TNBC cell lines. (**c**) Effects of *PICH* knockdown on cell growth of triple-negative (red) and ER/PR^+^ (blue) breast cancer cell lines. The indicated cells were infected with lentiviral particles for 6–8 days followed by imaging analyses and staining quantification. The left, middle, and right panels show the bright-field images, the crystal violet staining of cells, and the quantification of cell growth, respectively. The experiments were independently repeated three times in triplicate. Error bars represent means ± SD. *P*-values were calculated by two-tailed *t*-test comparing the control and treated group to each of the experimental groups. *P < 0.05, ***P < 0.001. **d**, **e** Effect of WT and ATPase-inactive PICH on impaired cell proliferation of TNBC cells induced by *PICH* knockdown: crystal violet staining of the plates (**d**) and its quantification (**e**). The indicated cells were infected with lentiviral particles for 7 days followed by crystal violet staining and quantification. The bar graph indicates means ± SD for three experiments. ***P < 0.001; ns, not significant. Student’s *t*-test

Since PICH is predominantly overexpressed in TNBC, we sought to determine whether PICH plays critical role in the proliferation of TNBC cells. We first analyzed a set of breast cancer cell lines that mirror the molecular subtypes of clinical tumors^36^ and found that the expression of *PICH* is much higher in a cohort of 23 TNBC cell lines than in a cohort of 28 Non-TNBC breast cancer cell lines (Fig. 2a). We verified this observation in a series of breast cancer cell lines, including two primary breast cancer cell lines obtained from the hospital, and confirmed that PICH levels are higher in TNBC cells than in luminal cells (Fig. 2b). Because the results obtained in the cell lines were consistent with the data obtained from primary tumors, we also concluded that these cell lines were able to provide an excellent platform to assess the potential roles of PICH in TNBC.

To investigate the specific role for PICH in cell proliferation, we used five TNBC cell lines and four ER^+^ breast cancer cell lines, including the two above mentioned primary breast cancer cell lines. We generated two distinct shRNAs to target PICH in various breast cancer cell lines, and both were found to efficiently reduce PICH expression (Supplementary Fig. S2A). We then generated TNBC and luminal breast cancer cell lines stably overexpressing shPICHs. The effect of *PICH* knockdown on cell proliferation was measured 6–8 days after virus infection. We found that the five TNBC cell lines (BT549, MDA-MB-468, MDA-MB-231, MDA-MB-436, and the primary cell line DFBC11–19) were extremely sensitive to *PICH* knockdown. In contrast, in the four luminal breast cell lines (MCF7, T47D, ZR-75–1, and the primary cell line DFBC4–17), *PICH* knockdown did not result in obvious inhibition on cell growth (Fig. 2c). As hypothesized, *PICH* knockdown strongly impaired the growth of all five TNBC cell lines tested.

To exclude off-target effects of the shRNAs, we carried out rescue experiments with both WT and ATPase inactive PICH mutant in TNBC cells, because PICH ATPase activity is very important for its function and centromere location in mitosis. We stably transfected WT and mutant PICH plasmids into MDA-MB-231 cells. The wild-type PICH was constrained to the KT region in prometaphase, while the mutant form was diffused among the chromosome arms (Supplementary Fig. S2B). The rescue effect of PICH on cell proliferation was measured 7 days after virus infection. Notably, we found that the proliferation of MDA-MB-231 cells expressing shPICH can be rescued by a wild-type resistant PICH (PICH-Res; Fig. 2d, e). However, expression of an ATPase inactive form of PICH-Res (WAB) failed to restore cell proliferation (Fig. 2d, e), indicating that ATPase activity of PICH is critical for the proliferation of TNBC cells.

### Downregulation of *PICH* expression induces apoptosis and impairs mitosis in TNBC cells

**Fig. 3:**
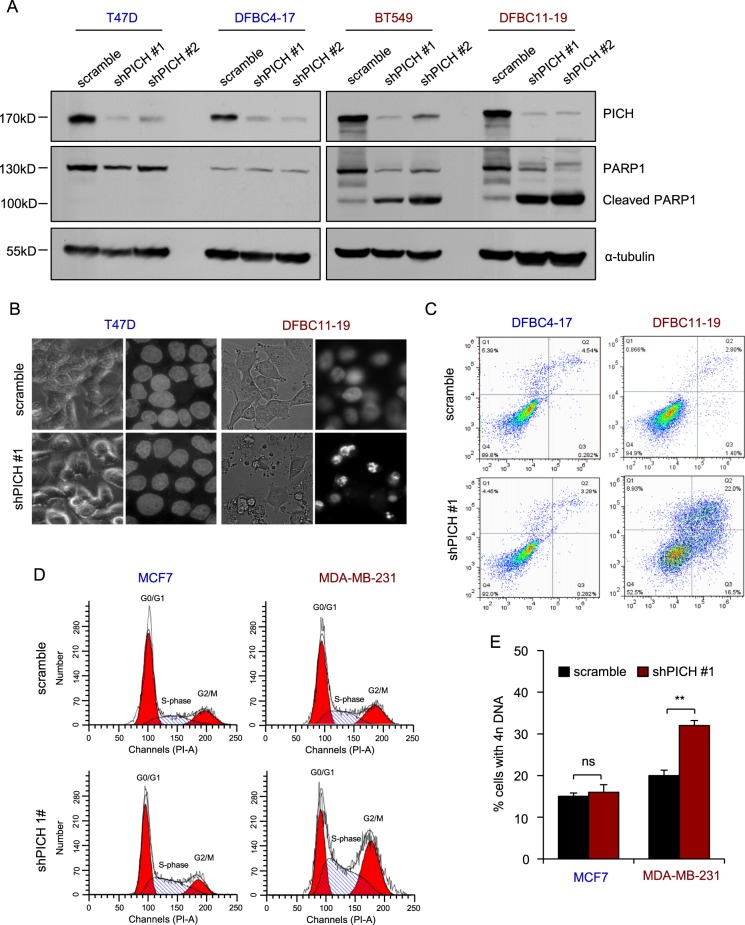
Downregulation of *PICH* expression induces apoptosis and impairs mitosis in TNBC cells. **a** Immunoblotting analysis for the indicated proteins in triple negative (BT549, DFBC11–19) and ER/PR^+^ (T47D, DFBC4–17) breast cancer cells overexpressing control shRNA or shPICH. **b** Effect of *PICH* knockdown on DNA fragmentation. The nuclei of DFBC11–19 and T47D cells overexpressing control shRNA or shPICH were stained with DAPI. The bright and punctate staining indicates DNA fragmentation. **c** Effect of *PICH* knockdown on the apoptosis of DFBC11–19 and DFBC4–17 primary breast cancer cells stained with an Annexin V detection kit. The percentage of apoptotic (Annexin V-positive) cells is indicated. **d** Effect of *PICH* knockdown on the accumulation of cells with 4n DNA content. MDA-MB-231 and MCF7 cells overexpressing control shRNA or shPICH were stained with PI for cell cycle analysis. The graph shows representative cell cycle histograms. **e** Quantification of the percentage of cells with 4n DNA upon *PICH* knockdown. The bar graph indicates means ± SD for three experiments. **P < 0.01; ns, not significant. Student’s *t*-test

To understand the mechanisms underlying PICH function in TNBC cells, we examined how PICH depletion affects various cellular processes. PICH knockdown had little effect on the apoptosis of luminal tumor cells, such as T47D and DFBC4–17, but exerted a pronounced effect on TNBC cells, exemplified by increased cleaved PARP1 and DNA fragmentation (Fig. 3a, b, Supplementary Fig. S3A). This observation was confirmed in apoptosis assays using Annexin V staining followed by flow cytometry (Fig. 3c, Supplementary Fig. S3B).

PICH is indispensable for efficient chromosome segregation. Therefore, we hypothesized that the cell death observed upon *PICH* inhibition might be due to a dysregulated cell cycle progression. In TNBC cells only, *PICH* knockdown induced an accumulation of cells with 4n DNA content (Fig. 3d, e, Supplementary Fig. S3C-D), indicating a G2/M arrest or failure of cytokinesis. Many studies have rectified that PICH inhibition does not affect the mitotic checkpoint activation, but influences the anaphase chromosome segregation. By immunofluorescence, we found that *PICH*-silenced TNBC cells exhibited no elevation of p-H3, a marker of G2/M (Supplementary Fig. S3E-F). Combining with these data, we speculated that *PICH* depletion may specifically regulate the division process in TNBC cells.

### *PICH* depletion causes chromosomal instability and cytokinesis failure in TNBC cells

**Fig. 4:**
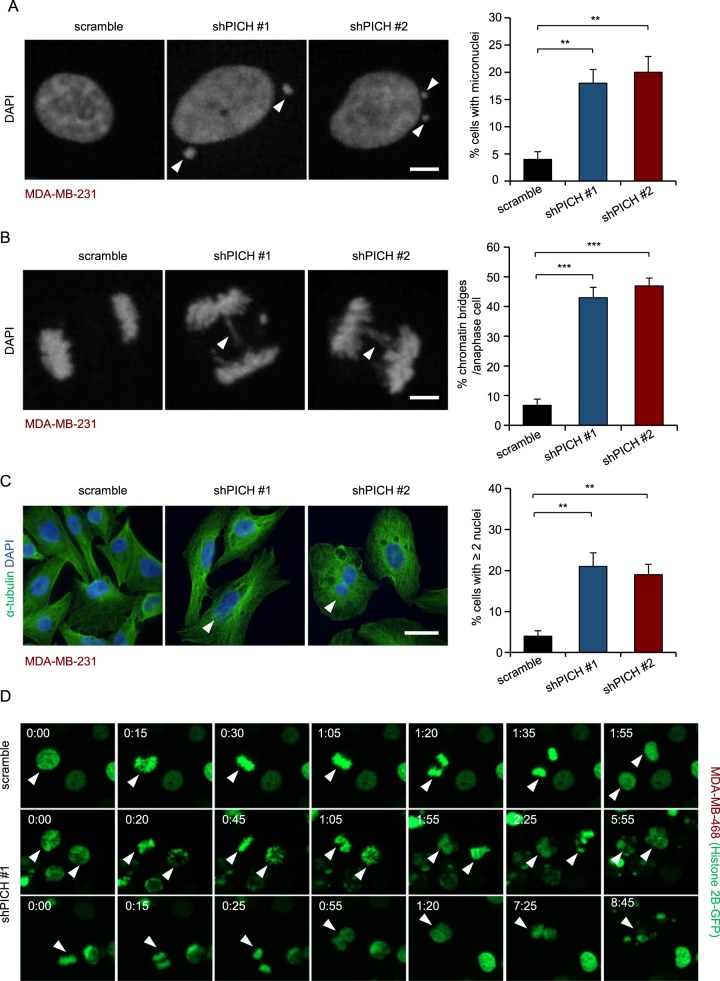
*PICH* depletion causes chromosomal instability and cytokinesis failure in TNBC cells. **a** Effect of *PICH* knockdown on micronucleus formation in asynchronously growing MDA-MB-231 cells. Puromycin-selected MDA-MB-231 cells were cultured for three days and stained with DAPI. Representative immunofluorescence images and quantification of micronuclei is shown. The white arrow indicates micronucleus. Scale bar, 5 μm. **b** Representative immunofluorescence images and frequency of chromatin bridge formation in anaphase cells stained with DAPI. MDA-MB-231 cells overexpressing control shRNA or shPICH were synchronized with thymidine (2 mM, sigma) for 24 h and released for 14 h to M phase. The cells were then fixed and stained with DAPI. Percentages of cells containing chromatin bridges in anaphase (*n* > 40 events per condition) were calculated. The white arrow indicates chromatin bridges. Scale bar, 5 μm. **c** Frequency of binucleation in asynchronously growing MDA-MB-231 cells stained with DAPI and alpha-tubulin. Representative images and quantification are shown. The white arrow indicates a binucleated cell. Scale bar, 15 μm. The bar graphs in (**a**–**c**) indicate means ± SD for three experiments. **P < 0.01 and *** *P* < 0.001, two-tailed student’s *t*-test. **d** MDA-MB-468 cells stably overexpressing GFP-H2B were infected with viruses overexpressing control shRNA or shPICH. After 4 days, the cells were subjected to time-lapse imaging (one image taken every 5 min). Time is given in hours:minutes

Because *PICH* knockdown in chicken and HeLa cells increases the occurrence of chromosomal abnormalities, such as chromatin bridges and micronuclei^27,28^, we analyzed whether *PICH*-deficient TNBC cells displayed an altered frequency of chromosomal abnormalities. We observed that *PICH*-silenced MDA-MB-231 cells had a significantly elevated frequency of micronucleus formation, chromatin bridges and binucleation, revealed by DAPI staining of nuclei or immunofluorescent staining for α-tubulin (Fig. 4a–c). *PICH*-silenced MDA-MB-436 cells also had higher percentage of binucleated cells than control cells (Supplementary Fig. S4C), while luminal *PICH*-depleted cells exhibited normal DNA morphology (Supplementary Fig. S4A-B). As a result, the increase of binucleation in TNBC cells leads to accumulation of cells with 4n DNA content. We next used time-lapse microscopy of GFP-Histone 2B (H2B)-expressing MDA-MB-468 cells to determine whether apoptosis and defective mitosis due to *PICH* knockdown were functionally associated. Cell death events were dramatically increased upon *PICH* knockdown (3 out of 216 cells in control, 308 out of 387 in *PICH*-silenced cells during the 24 h of imaging). Moreover, cell death events were often followed by division abnormalities in the *PICH*-silenced population: cells with double nuclei, which had failed cytokinesis, often underwent cell death; additionally, some cells with an apparently abnormal metaphase plate were unable to progress towards anaphase, and underwent cell death directly after mitosis (Fig. 4d). Together, these data suggest that TNBC cells depend on PICH for proper mitosis; inhibition of PICH in these cells causes impaired mitosis and consequent cell death.

### *PICH* silencing suppresses TNBC tumor growth in vivo

**Fig. 5:**
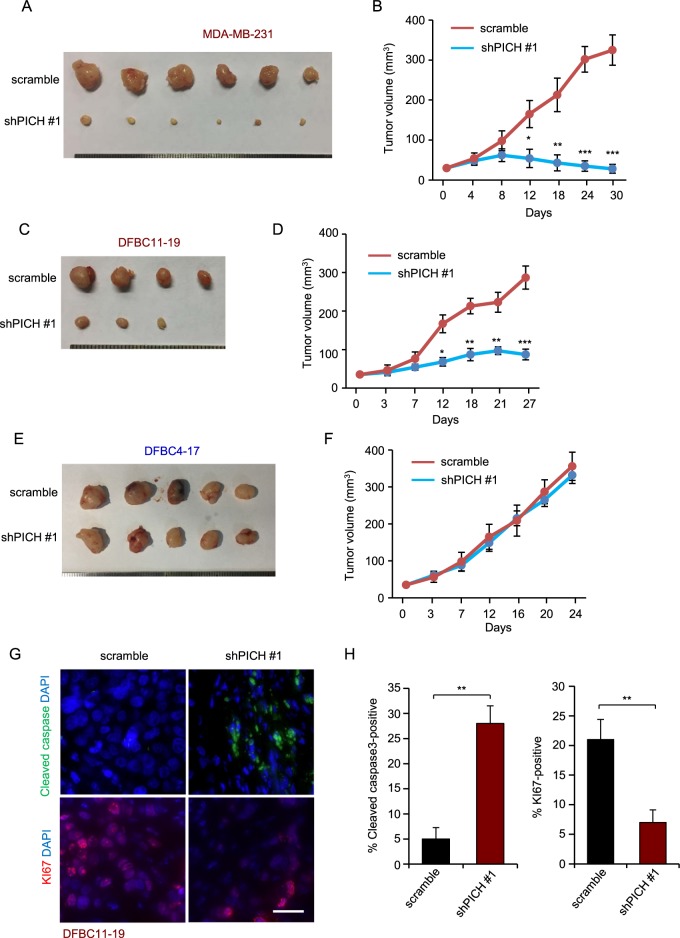
*PICH* silencing suppresses tumor growth in vivo. **a**, **c**, **e** MDA-MB-231, DFBC11–19 and DFBC4–17 cells stably overexpressing control shRNA or shPICH were cultured for 2 days, and then injected into the mammary fat pad of nude mice. Tumor masses were harvested after they had grown for about 4 weeks. **b**, **d**, **f** Effect of *PICH* knockdown on tumor volume. Tumors were measured every 3–6 days in two dimensions with a caliper. Error bars represent means ± SEM. *P*-values were calculated with the two-tailed Student’s t-test. *P < 0.05, **P < 0.01 and *** *P* < 0.001. **g**, **h** The harvested DFBC11–19 tumors were fixed and stained for KI67 and cleaved caspase3. Representative micrographs (**e**) and quantification (**f**) of the staining (*n* = 3 mice in each treatment group). Scale bar, 25 µm. Error bars represent means ± SEM. *P*-values were calculated with the two-tailed Student’s *t*-test. **P < 0.01

To determine whether PICH is also important for TNBC growth in vivo, we used the invasive breast cancer cell line MDA-MB-231 in xenograft tumor experiments in nude mice. We transplanted TNBC cells expressing control shRNA or shPICH into the mammary fat pads of nude mice to allow orthotopic tumor formation. All recipient mice developed tumors within one month in the control group; however, mice in the *PICH*-depleted group failed to develop tumors (Fig. 5a, b). We further validated the anti-proliferative effect of *PICH* inhibition in the primary TNBC cells DFBC11–19. *PICH* knockdown also decreased tumor growth (Fig. 5c, d). These results demonstrated that knockdown of *PICH* strikingly suppresses the xenograft growth, suggesting that PICH is required for the proliferation of TNBC cells in vivo. We then collected and fixed the tumor of xenografts derived from the primary TNBC cells. *PICH*-knockdown tumors showed a significant decrease in the number of the proliferating cells (Ki67-positive) and a great increase in the number of apoptotic cells (cleaved caspase3-positive; Fig. 5g, h). These data demonstrated that *PICH* silencing dramatically inhibits TNBC cells proliferation and induces apoptosis in vivo. In contrast, *PICH* knockdown did not inhibit tumor growth and proliferation rate in the primary luminal cancer cells DFBC4–17 (Fig. 5e, f, Supplementary Fig. S5A-B). Together, these data indicate the PICH is selectively required for the oncogenic growth of TNBC cells, providing the evidence that PICH inhibition could be an effective approach in treating TNBC.

## Discussion

Patients with TNBC have limited treatment options due to the aggressive features of the disease and the current lack of validated effective molecular targets^3,7–9^. In this study, we analyzed PICH expression by IHC on clinical samples and found that PICH is highly expressed in breast cancer lacking the expression of ER/PR/HER2. Remarkably, we interrogated multiple datasets and concluded that high expression of PICH correlates with higher risk of distal metastasis and decreased overall survival. We also demonstrated that silencing of *PICH* expression in human TNBC cell lines significantly inhibits cell proliferation by inducing apoptosis. However, *PICH* knockdown did not exert this effect on luminal cancer cells. *PICH* knockdown can also inhibit TNBC cells growth in xenografts and importantly, in established primary TNBC tumors. Remarkably, the ATPase activity of PICH is required for the survival and proliferation of TNBC cells. Thus, our data show that PICH is important for the growth and survival of TNBC and is a potential novel therapeutic target in TNBC.

The prognostic value of PICH expression is likely due to its correlation with cell cycle and proliferation. PICH was first identified as a mitotic binding partner and substrate of PLK1, a critical kinase for mitotic progression^24^. Knockdown of *PLK1* causes PICH mislocalization from the centromeric region to the chromosome arms, resembling the phenotype of PICH ATPase mutant^24^. Previous studies have demonstrated that PLK1 is significantly overexpressed in TNBC compared with the other breast cancer subtypes^37^. We thus speculate that PLK1 regulates PICH localization and activity in TNBC cells, ensuring the precise execution of bridge resolution and chromosome segregation. Interestingly, the analysis of breast cancer datasets revealed that the expression of *PICH* correlates with that of mitosis-related genes, such as *KIF4A*, *CEP55*, *MKI67*, *MELK*, *BUB1*, *CENPA*, and *AURKB* (Supplementary Table 2). Notably, these genes are heavily enriched in the basal-like 1 TNBC subtype^38^ and are also major components of a multi-gene signature for predicting disease outcome^39^.

Many studies have revealed that the resolution of PICH-positive threads requires topoisomerase IIa (TOP2A*)* activity. PICH and TOP2A functionally cooperate to prevent chromosome missegregation events in mitosis^28,40^. Surprisingly, *TOP2A* expression is also significantly more frequent in patients with TNBC^41,42^. TNBC is currently treated with chemical drugs such as taxanes, alkylating agents, and platinum agents^8,13^. TOP2A is essential during mitosis for proper segregation of daughter chromosomes and is a target of anthracyclines^28,43^. PICH can strongly stimulate the catalytic activity of TOP2A in vitro and its deficiency partially resembles inhibition of the TOP2A decatenation activity^28^. Moreover, PICH ATPase activity is indispensible for the proper function in TNBC cells, and we also observed that wild-type PICH but not ATPase mutants accumulates at kinetochores during mitosis in TNBC cells, so decreasing PICH expression or inhibition its activity in TNBC cells may promote the sensitivity to TOP2A inhibitors. PICH deficiency causes persisted chromatin bridges in the midzone, which triggers cleavage furrow regression, leading to cytokinesis failure and binucleation^28^. Therefore, we propose that PICH is a sensor of streched bridge DNA and serves as a recruitment platform for other factors, such as BLM, Topo IIa and Topo III, ensuring faithful chromosome segregation and rapid proliferation in TNBC cells. Future studies will aim to investigate whether the hypothesis is the molecular mechanism of how PICH regulates chromosomal instability and cytokinesis failure in TNBC cells.

Notably, we showed that PICH overexpression was closely related to high risk of recurrence and distal metastasis. Since most patients with TNBC are prone to have a high rate of local and systemic relapse after traditional chemotherapy, it is important to identify more effective targeted treatments for TNBC. Pich is highly expressed in mouse embryonic development and its loss at that stage results in chromosomal instability;^29,44,45^ however, its expression dramatically decreases afterwards^44^. By analyzing clinical tissue samples, we showed that PICH had very low expression in normal breast tissue. Therefore, PICH is an excellent therapeutic target for TNBC.

However, why PICH is selectively overexpressed and required for bridge resolution in TNBC cells and not in other types of breast cancer cells remains an open question. Due to the intrinsic genetic heterogeneity, TNBCs have more frequent p53 mutations, higher mitotic index, and phenotypical similarity to BRCA1-deficient breast cancers^4,10,13–15^. The genome guardian p53 acts as a checkpoint in the cell-cycle to trigger molecular responses to DNA damage, including repair and apoptosis. Mutant p53 can promote a range of centrosome abnormalities to drive mitotic defects and multinucleation^46,47^. Further, studies have revealed that PARP1 inhibitor olaparib, a candidate clinical drug for TNBC, induces cytotoxicity leading to mitotic DNA damage, finally causing cytokinesis failure, multinucleation and cell death^20^. Therefore, we hypothesized that TNBC cells are more susceptible to death because of genetic aberrance in mitosis. Accordingly, PICH may play this role uniquely and is selectively overexpressed in these cells. In luminal breast cancer cells, PICH still functions during division, but it may not be essential due to redundancy with other related ATPases. Additional studies are required to determine the precise regulation of PICH overexpression in TNBC cells, and the reason for its selective role.

In summary, we found that PICH has a novel and fundamental role in TNBC. TNBCs express high levels of PICH, and the high expression of this molecule predicts poor outcome. Depletion of PICH in TNBC cells causes cytokinesis failure, binucleation and apoptosis. Thus, we suggest that PICH may be a promising therapeutic target in combination with conventional chemotherapy, in particular for the treatment of patients with TNBC.

## Materials and methods

### Patient data

Patient survival and gene expression data for independent cohorts of patients with breast cancer were accessed from the Breast Cancer Integrative platform (BCIP; http://www.omicsnet.org/bcancer/)^48^, an open source, gene-centered platform for identifying potential regulatory genes in breast cancer. For PICH immunohistochemical staining, we obtained 194 formalin-fixed and paraffin-embedded tumor specimens from a tissue bank at the Department of Pathology, Affiliated Hospital of Third Military Medical University, Chongqing, China. All tumors were primary and untreated, and we obtained detailed clinicopathological information for all tumors, including immunohistochemical staining for ER-α, PR, and HER2. Informed consent was obtained from all subjects or their relatives.

### IHC staining, immunofluorescence, and Western blotting

We performed IHC staining for PICH, using an anti-PICH antibody diluted with 1:100 on sections that were 4-µm thick; the detailed method is in a study previously published by our lab^49^. The staining was assessed by pathologists blinded to the origin of the samples. The German semi-quantitative scoring system was used to evaluate the intensity and extent of the staining. Each specimen was assigned a score according to the intensity (no staining = 0, weak staining = 1, moderate staining = 2, strong staining = 3) and proportion of stained cells (0% = 0, 1–24% = 1, 25–49% = 2, 50–74% = 3, 75–100% = 4). The final immunoreactive score was determined by multiplying the intensity score by its extent, ranging from 0 (minimum score) to 12 (maximum score).

Cells were fixed with 4% formaldehyde for 10 min. After washing, cells were permeablized with 0.1% Trition X-100/PBS for 10 min. Cells were then blocked with 3% normal goat serum in 0.1% Triton X-100/PBS for 1 h at room temperature. Primary antibodies were added in blocking buffer overnight at 4 °C and then incubated with secondary antibodies at room temperature for 1 h. All images were taken with a 60×/NA 1.42 oil objective (Olympus, Japan) on a DeltaVision Restoration Microscope (General Electric Company, American). Volocity 6.0 software was used for analysis of the images, which includes merging channels with different colors and cropping.

Cultured cells were lysed in RIPA buffer (50 mM Tris, pH 7.4, 150 mM NaCl, 1% Nonidet P-40, 0.5% sodium deoxycholate, and 0.1% sodium dodecyl sulfate), which was supplemented with protease inhibitors cocktail (Roche) and phosphatase inhibitors cocktail (Thermo Scientific). After incubation, lysates were cleared by centrifugation. Proteins were separated by SDS–PAGE and analyzed by western blotting with the indicated antibodies.

### Plasmid construction and antibodies

A plasmid overexpressing a GFP-tagged human PICH was kindly provided by Professor Hongtao Yu from University of Texas Southwestern Medical Center, Dallas, USA^27^. We generated a mutant (PICH-WAB) in which residues K128 (Walker A) and E229 (Walker B) were substituted by alanine (A) and glutamine (Q) and shPICH-resistant PICH with silent mutations via Quickchange XL Site-directed Mutagenesis (Stratagene, La Jolla, CA, USA). To generate pLKO-shRNA plasmids, overexpressing short hairpin RNAs (shRNA) targeting human *PICH*, oligonucleotides were designed and synthesized (Invitrogen). Following annealing, the double-stranded oligonucleotides were ligated into the pLKO vector digested with AgeI and EcoRI. The sequences for the primers used for the mutagenesis, scramble, shPICH #1 and shPICH #2 are listed in Supplementary Table 1.

The PICH antibody used for western blotting was also donated by professor Yu and the PICH antibody for immunohistochemistry staining was bought from the sigma company (Sigma; 1:100, Cat#HPA050492). For immunostaining, rabbit anti-KI67 polyclonal antibody (Abcam; 1:200, Cat#ab16667), rabbit anti-cleaved caspase3 polyclonal antibody (Cell signalling technology; 1:100, Cat#9664), rabbit anti-p-H3 polyclonal antibody (Cell signalling technology; 1:100, Cat#53348), mouse anti-α-tubulin monoclonal antibody (Sigma; 1:200, Cat#T5168), Human anti-centromere CREST antibody (Antibodies Incorporated; 1:500, Cat#: 15–234), DAPI (Invitrogen) and cross-adsorbed secondary antibodies from Molecular Probes were used. The primary antibodies used for Western blotting were mouse anti-α-tubulin monoclonal antibody (Sigma; 1:5000, Cat#T5168), rabbit anti-PARP1 polyclonal antibody (Cell signalling technology; 1:1000, Cat#9542).

### Cell culture, transfection, and infection

Human breast cancer cell lines (MCF7, T47D, ZR-75–1, BT549, HCC38, MDA-MB-361, MDA-MB-453, SK-BR-3, MDA-MB-231, MDA-MB-436, and MDA-MB-468) were bought from the American Type Culture Collection. The breast cancer cell lines MCF7, T47D, ZR-75–1, MDA-MB-453, SK-BR-3, MDA-MB-468 were cultured in DMEM, 10% fetal bovine serum (FBS), and 1% penicillin/streptomycin. The breast cancer cell lines BT549, HCC38, MDA-MB-361, MDA-MB-231, MDA-MB-436 were cultured in RPMI-1640, 10% FBS, and 1% penicillin/streptomycin. The primary breast cancer cell lines DFBC11–19 (ER^-^PR^-^HER2^-^) and DFBC4–17 (ER^+^PR^-^HER2^+^) were provided by the Tianjin Medical University Cancer Institute and Hospital (Tianjin, China) and were cultured in DMEM-F12, 10% FBS, and 1% penicillin/streptomycin. For packaging the viruses, HEK293T cells from the American Type Culture Collection were grown in DMEM with 10% FBS and 1% penicillin/streptomycin.

Retroviruses were prepared by transient co-transfection with packaging DNA plus lenti-pLKO vectors into HEK293T cells. Typically, 6 μg vector DNA, 4.5 μg psPAX2, 1.5 μg pMD2-VSVG, and 24 μL Lipofectamine 2000 (Thermo Scientific) were used. DNA and lipids were pre-diluted in 500 μL Opti-MEM™ (invitrogen) individually and then mixed. After 15 min of incubation, the DNA-lipid mixtures were added to HEK293T cells. Viral supernatant was collected two and three days after transfection, filtered through 0.45-μm filters, and added at a multiplicity of infection (MOI) of 5 to the target cells in the presence of polybrene (8 μg/mL, Millipore). Puromycin (1–2.5 μg/mL, sigma) was used to treat different cells for two days, to kill cells that had not been infected. The remaining of the cells were cultured and used as indicated.

### Proliferation, apoptosis, and DNA content assays

Cells were infected with lentiviral particles, subjected to puromycin selection, harvested, and seeded in 12-well plates. Cells were fixed with 4% formaldehyde 4–6 days after seeding and stained with crystal violet. The plates were washed extensively and imaged with a flatbed scanner. For quantification of the staining, 1 mL 10% acetic acid was added to each well to solubilize the dye. The absorbance was measured at 590 nm with 750 nm as a reference (Infinite M1000, Tecan).

Apoptosis was evaluated by flow cytometry analysis using an Annexin V apoptosis detection kit (eBioscience, USA) according to manufacturer’s protocol. DNA fragmentation was analyzed by DAPI staining and PARP1 cleavage by western blotting. Analysis of DNA content with flow cytometry was performed using propidium iodide (PI, Sigma) staining with standard procedures. Samples were analyzed on a FACSCalibur (BD Biosciences) flow cytometer.

### Time-lapse imaging

Time-lapse imaging was performed on an inverted microscope (Nikon Eclipse Ti-E), which was equipped with a Perfect Focus System and a humidified incubation chamber (37 °C, 5% CO_2_). Puromycin-selected MDA-MB-468 cells stably expressing GFP-H2B were pre-seeded in eight-chambered cover glasses (Nunc Lab-Tek, Thermo Scientific), and then cultured for two days. Images were captured every five minutes for 24 h, using a 20× objective lens. Images were analyzed using the Volocity software, and cell behavior was analyzed manually.

### Tumor xenograft studies

The recipient mice were 6-weeks old NCR-nude female mice (Vital River, Beijing). All xenograft studies were performed with the approval of the Institutional Animal Care and Use Committee of our institution. Cells were resuspended in PBS and placed on ice until injection. Mice were anesthetized by inhalation of isoflurane, and injected in the mammary fat pad with 100 μL cell suspension (5 × 10^6^ cells) per site. Tumors were measured every 3–6 days in two dimensions with a caliper. Tumor volume was calculated using the formula: *V* = 0.5 × length × width × width. All xenograft data are presented as mean ± SEM. All mice were sacrificed at the end of the experiment and tumors were harvested and fixed for immunofluorescence staining.

### Statistical analysis

Statistical analysis were performed using the GraphPad Prism version 6.0. Student’s *t*-test or Mann–Whitney *U* test was used for differential comparison between two groups. The log-rank test was used to determine *P* values for all Kaplan–Meier survival curve analyses. The correlations between the expression levels of *PICH* and other genes in patients with breast cancer were analyzed by calculating Pearson’s correlation coefficient.

## Supplementary information

Supplemental material

## References

[1] Brenton JD, Carey LA, Ahmed AA, Caldas C (2005). Molecular classification and molecular forecasting of breast cancer: ready for clinical application?. J. Clin. Oncol..

[2] Nielsen TO (2004). Immunohistochemical and clinical characterization of the basal-like subtype of invasive breast carcinoma. Clin. Cancer Res..

[3] Perou CM (2000). Molecular portraits of human breast tumours. Nature.

[4] Sorlie T (2001). Gene expression patterns of breast carcinomas distinguish tumor subclasses with clinical implications. Proc. Natl Acad Sci. USA.

[5] Konecny G (2003). Quantitative association between HER-2/neu and steroid hormone receptors in hormone receptor-positive primary breast cancer. J. Natl. Cancer Inst..

[6] Slamon DJ (1989). Studies of the HER-2/neu proto-oncogene in human breast and ovarian cancer. Science.

[7] Rakha EA, Reis-Filho JS, Ellis IO (2008). Basal-like breast cancer: a critical review. J. Clin. Oncol..

[8] Carey LA (2007). The triple negative paradox: primary tumor chemosensitivity of breast cancer subtypes. Clin. Cancer Res..

[9] Banerjee S (2006). Basal-like breast carcinomas: clinical outcome and response to chemotherapy. J. Clin. Pathol..

[10] Foulkes WD, Smith IE, Reis-Filho JS (2010). Triple-negative breast cancer. N. Engl. J. Med..

[11] Bianchini G, Balko JM, Mayer IA, Sanders ME, Gianni L (2016). Triple-negative breast cancer: challenges and opportunities of a heterogeneous disease. Nat. Rev. Clin. Oncol..

[12] Dent R (2007). Triple-negative breast cancer: clinical features and patterns of recurrence. Clin. Cancer Res..

[13] Cleator S, Heller W, Coombes RC (2007). Triple-negative breast cancer: therapeutic options. Lancet Oncol..

[14] Lakhani SR (2002). The pathology of familial breast cancer: predictive value of immunohistochemical markers estrogen receptor, progesterone receptor, HER-2, and p53 in patients with mutations in BRCA1 and BRCA2. J. Clin. Oncol..

[15] Sorlie T (2003). Repeated observation of breast tumor subtypes in independent gene expression data sets. Proc. Natl Acad Sci. USA.

[16] Turner N, Tutt A, Ashworth A (2004). Hallmarks of ‘BRCAness’ in sporadic cancers. Nat. Rev. Cancer.

[17] Farmer H (2005). Targeting the DNA repair defect in BRCA mutant cells as a therapeutic strategy. Nature.

[18] Turner N, Tutt A, Ashworth A (2005). Targeting the DNA repair defect of BRCA tumours. Curr. Opin. Pharmacol..

[19] Murai J (2012). Trapping of PARP1 and PARP2 by Clinical PARP Inhibitors. Cancer Res..

[20] Schoonen PM (2017). Progression through mitosis promotes PARP inhibitor-induced cytotoxicity in homologous recombination-deficient cancer cells. Nat. Commun..

[21] Torres K, Horwitz SB (1998). Mechanisms of Taxol-induced cell death are concentration dependent. Cancer Res..

[22] Jordan MA (1996). Mitotic block induced in HeLa cells by low concentrations of paclitaxel (Taxol) results in abnormal mitotic exit and apoptotic cell death. Cancer Res..

[23] Morse DL, Gray H, Payne CM, Gillies RJ (2005). Docetaxel induces cell death through mitotic catastrophe in human breast cancer cells. Mol. Cancer Ther..

[24] Baumann C, Korner R, Hofmann K, Nigg EA (2007). PICH, a centromere-associated SNF2 family ATPase, is regulated by Plk1 and required for the spindle checkpoint. Cell.

[25] Kurasawa Y, Yu-Lee LY (2010). PICH and cotargeted Plk1 coordinately maintain prometaphase chromosome arm architecture. Mol. Biol. Cell.

[26] Kaulich M, Cubizolles F, Nigg EA (2012). On the regulation, function, and localization of the DNA-dependent ATPase PICH. Chromosoma.

[27] Ke Y (2011). PICH and BLM limit histone association with anaphase centromeric DNA threads and promote their resolution. EMBO J..

[28] Nielsen CF (2015). PICH promotes sister chromatid disjunction and co-operates with topoisomerase II in mitosis. Nat. Commun..

[29] Albers E (2018). Loss of PICH Results in Chromosomal Instability, p53 Activation, and Embryonic Lethality. Cell Rep..

[30] Pu SY (2017). ERCC6L, a DNA helicase, is involved in cell proliferation and associated with survival and progress in breast and kidney cancers. Oncotarget.

[31] Atlas N, Cancer Genome (2012). Comprehensive molecular portraits of human breast tumours. Nature.

[32] Curtis C (2012). The genomic and transcriptomic architecture of 2,000 breast tumours reveals novel subgroups. Nature.

[33] Bos PD (2009). Genes that mediate breast cancer metastasis to the brain. Nature.

[34] Hatzis C (2011). A genomic predictor of response and survival following taxane-anthracycline chemotherapy for invasive breast cancer. JAMA.

[35] Schmidt M (2008). The humoral immune system has a key prognostic impact in node-negative breast cancer. Cancer Res..

[36] Neve RM (2006). A collection of breast cancer cell lines for the study of functionally distinct cancer subtypes. Cancer Cell.

[37] Maire V (2013). Polo-like kinase 1: a potential therapeutic option in combination with conventional chemotherapy for the management of patients with triple-negative breast cancer. Cancer Res..

[38] Lehmann BD (2011). Identification of human triple-negative breast cancer subtypes and preclinical models for selection of targeted therapies. J. Clin. Invest..

[39] Venet D, Dumont JE, Detours V (2011). Most random gene expression signatures are significantly associated with breast cancer outcome. PLoS Comput. Biol..

[40] Rouzeau S (2012). Bloom’s syndrome and PICH helicases cooperate with topoisomerase IIalpha in centromere disjunction before anaphase. PLoS ONE.

[41] Mrklic I, Pogorelic Z, Capkun V, Tomic S (2014). Expression of topoisomerase II-alpha in triple negative breast cancer. Appl. Immunohistochem. Mol. Morphol..

[42] Nakagawa M (2011). Expression of p53, Ki-67, E-cadherin, N-cadherin and TOP2A in triple-negative breast cancer. Anticancer Res..

[43] Mordente A, Meucci E, Martorana GE, Tavian D, Silvestrini A (2017). Topoisomerases and anthracyclines: recent advances and perspectives in anticancer therapy and prevention of cardiotoxicity. Curr. Med. Chem..

[44] Yin Y, Tang L, Zhang J, Tang B, Li Z (2011). Molecular cloning and gene expression analysis of Ercc6l in Sika deer (Cervus nippon hortulorum). PLoS ONE.

[45] Xu Y, Chen X, Li Y (2005). Ercc6l, a gene of SNF2 family, may play a role in the teratogenic action of alcohol. Toxicol. Lett..

[46] Fujiwara T (2005). Cytokinesis failure generating tetraploids promotes tumorigenesis in p53-null cells. Nature.

[47] Noll JE (2012). Mutant p53 drives multinucleation and invasion through a process that is suppressed by ANKRD11. Oncogene.

[48] Wu J (2017). BCIP: a gene-centered platform for identifying potential regulatory genes in breast cancer. Sci. Rep..

[49] Pan X (2011). Elevated expression of CUEDC2 protein confers endocrine resistance in breast cancer. Nat. Med..

