# Supplementary material for: Loss of PICH promotes chromosome instability and cell death in triple-negative breast cancer
Source: Cell Death Dis. 2019 Jun 3;10(6):428. doi: 10.1038/s41419-019-1662-6 (PMC6547724; doi:10.1038/s41419-019-1662-6)
Supplement: Supplementary file 1 — Supplemental material [file 41419_2019_1662_MOESM1_ESM.pdf]

Supplementary Materials

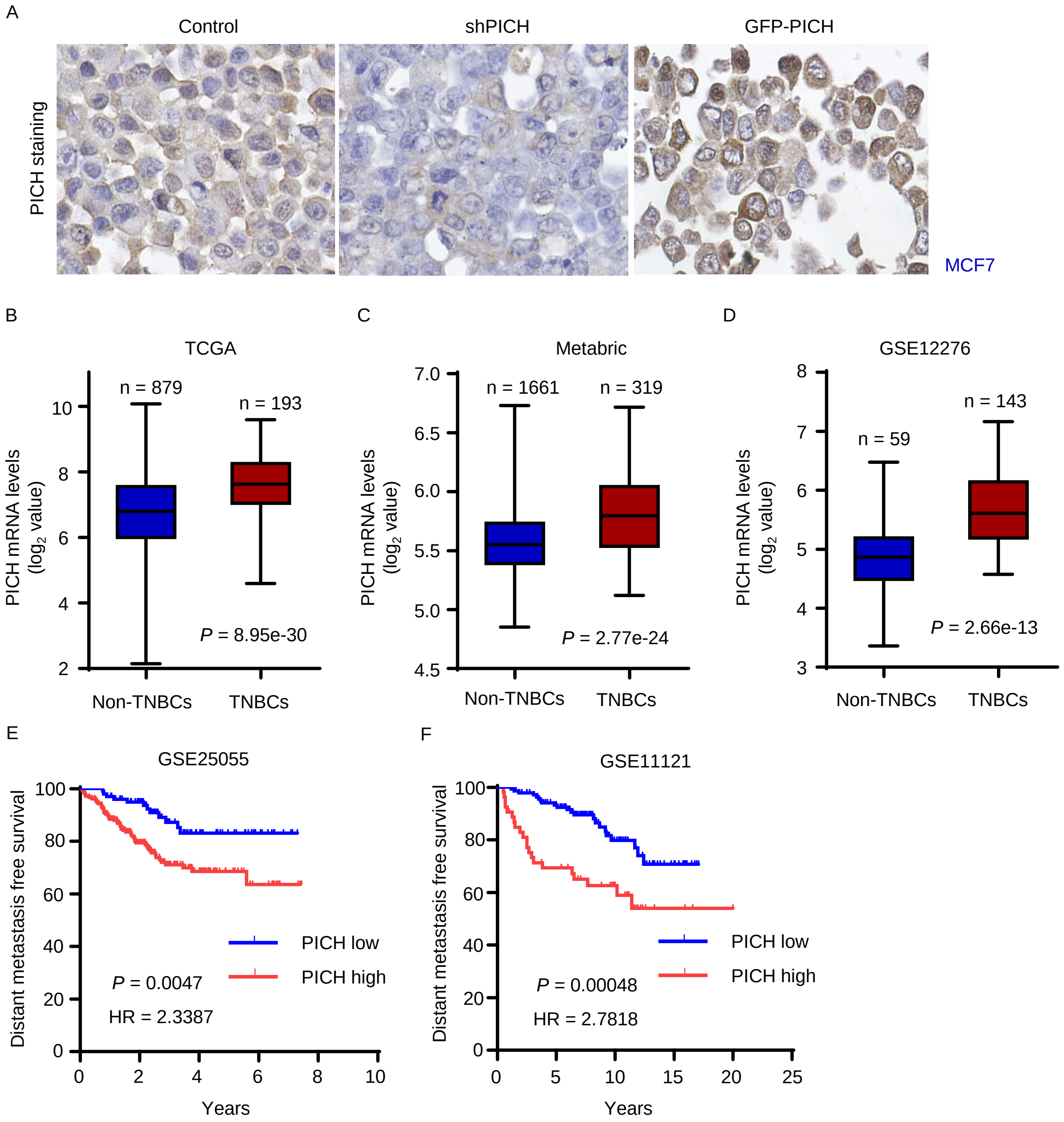

Figure S1. ***PICH*** **expression is upregulated in TNBC cohorts and correlates with distal metastasis**

(A) Specificity of the anti-PICH antibody by IHC. MCF7 cells were infected with shPICH- or GFP-PICH-overexpressing lentiviruses and stained for PICH. (B-D) *PICH* gene expression in the TCGA Breast, METABRIC and GSE12276 datasets. The cohorts in the datasets were divided into non-TNBCs (blue) and TNBCs (red) according to their receptors' expression status. Gene expression is reported as median-centered expression $\log_2$ values. The number of patients (n) per group is indicated. *P*-values were determined using a Mann-Whitney U test. (E) Kaplan–Meier analysis of metastasis-free survival of patients with breast cancer in two independent cohorts (GSE25055, GSE11121). Samples were divided into two groups with high and low expression levels of PICH. *P*-values were obtained from the log-rank test.

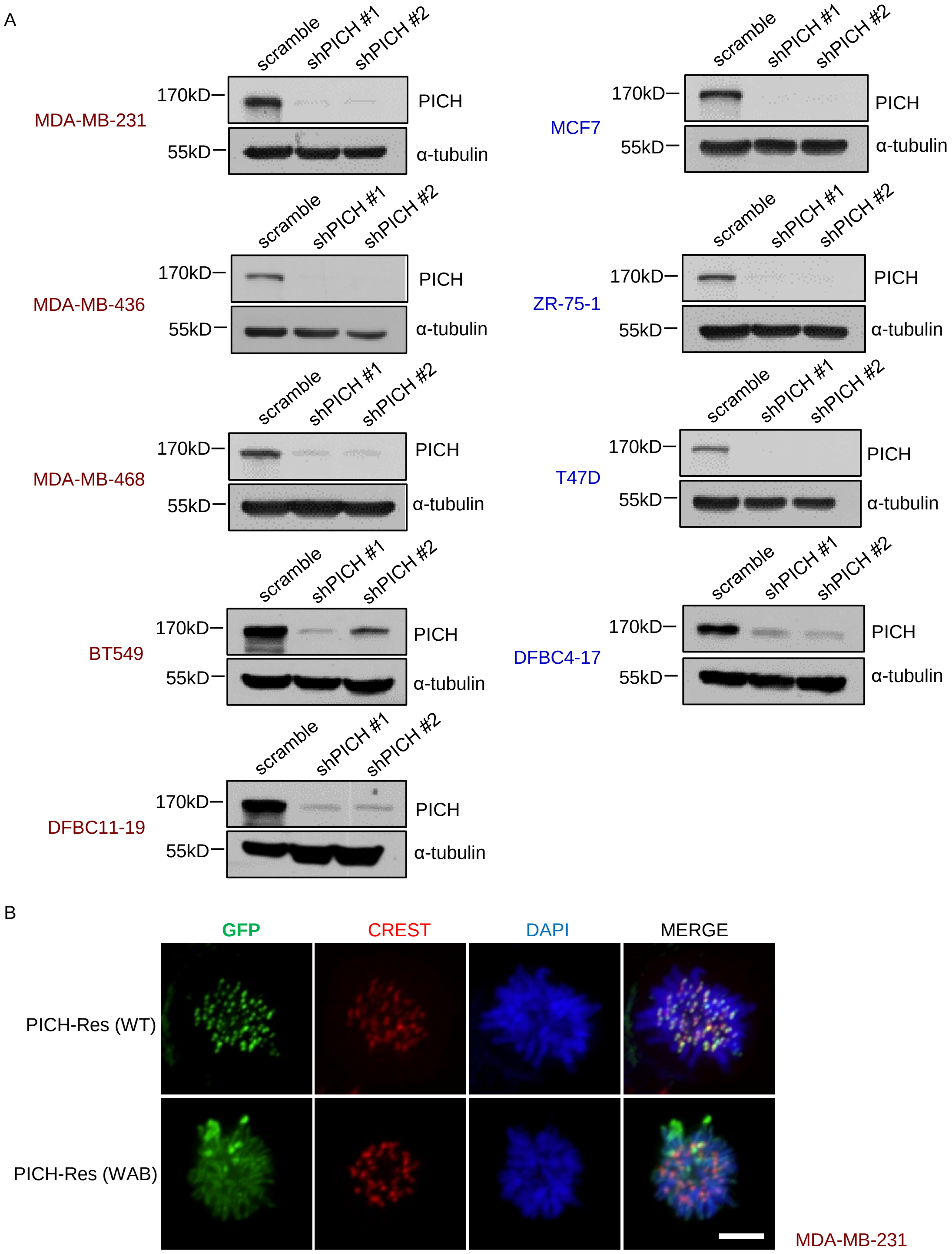

Figure S2. Efficiency of *PICH* knockdown in various cell lines and the cellular localization of silencing-resistant PICH

(A) Immunoblotting analysis of *PICH* knockdown effect in various breast cancer cell lines; α-tubulin was used as a loading control. (B) To rescue the *PICH* knockdown effect, MDA-MB-231 cells were infected with lentivirus stably overexpressing shPICH #1-resistant GFP-tagged PICH (WT or WAB mutants). Representative images of PICH (WT or WAB mutants) location durning mitosis. Scale bar, 5 μm.

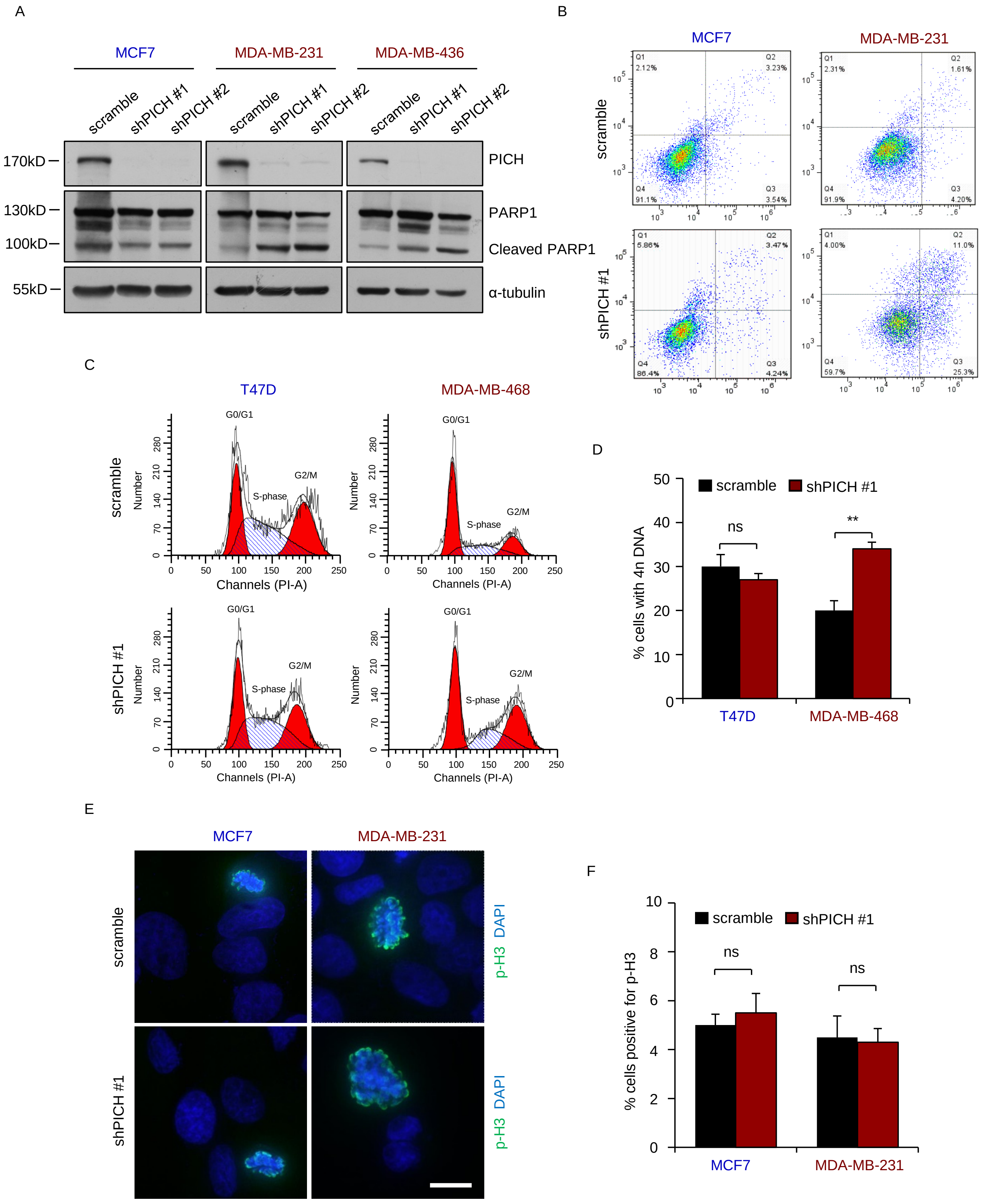
A
MCF7
MDA-MB-231
MDA-MB-436
scramble
shPICH #1
shPICH #2
170kD
130kD
100kD
55kD
PICH
PARP1
Cleaved PARP1
α-tubulin
B
scramble
shPICH #1
C
T47D
MDA-MB-468
G0/G1
S-phase
G2/M
Number
Channels (PI-A)
D
% cells with 4n DNA
ns
**
E
p-H3 DAPI
F
% cells positive for p-H3

Figure S3. ***PICH*** **depletion induces apoptosis and impairs mitosis in TNBC cells**

(A) Immunoblotting analysis of indicated proteins of ER/PR^{+} (MCF7) and triple negative (MDA-MB-231, MDA-MB-436) breast cancer cells overexpressing control shRNA or shPICH. (B) Effect of *PICH* knockdown on cell apoptosis in MCF7 and MDA-MB-231 breast cancer cells that were stained with Annexin V. The percentage of apoptotic (Annexin V-positive) cells is indicated. (C-D) Effect of *PICH* knockdown on the accumulation of cells with 4n DNA content. T47D and MDA-MB-468 cells overexpressing control shRNA or shPICH were stained with PI for cell cycle analysis. The graph shows representative cell cycle histograms. The percentage of 4n DNA cells is presented in (D). (E-F) Effect of *PICH* knockdown on the proportion of M phase cells. MCF7 and MDA-MB-231 cells were stained with p-H3, a marker for mitotic cells (E). Scale bar, 15 μm. The quantification of cells in M phase is in (F). The bar graph in (D) and (F) indicate means ± SD for three experiments. **P < 0.01; ns, not significant. Student's t-test.

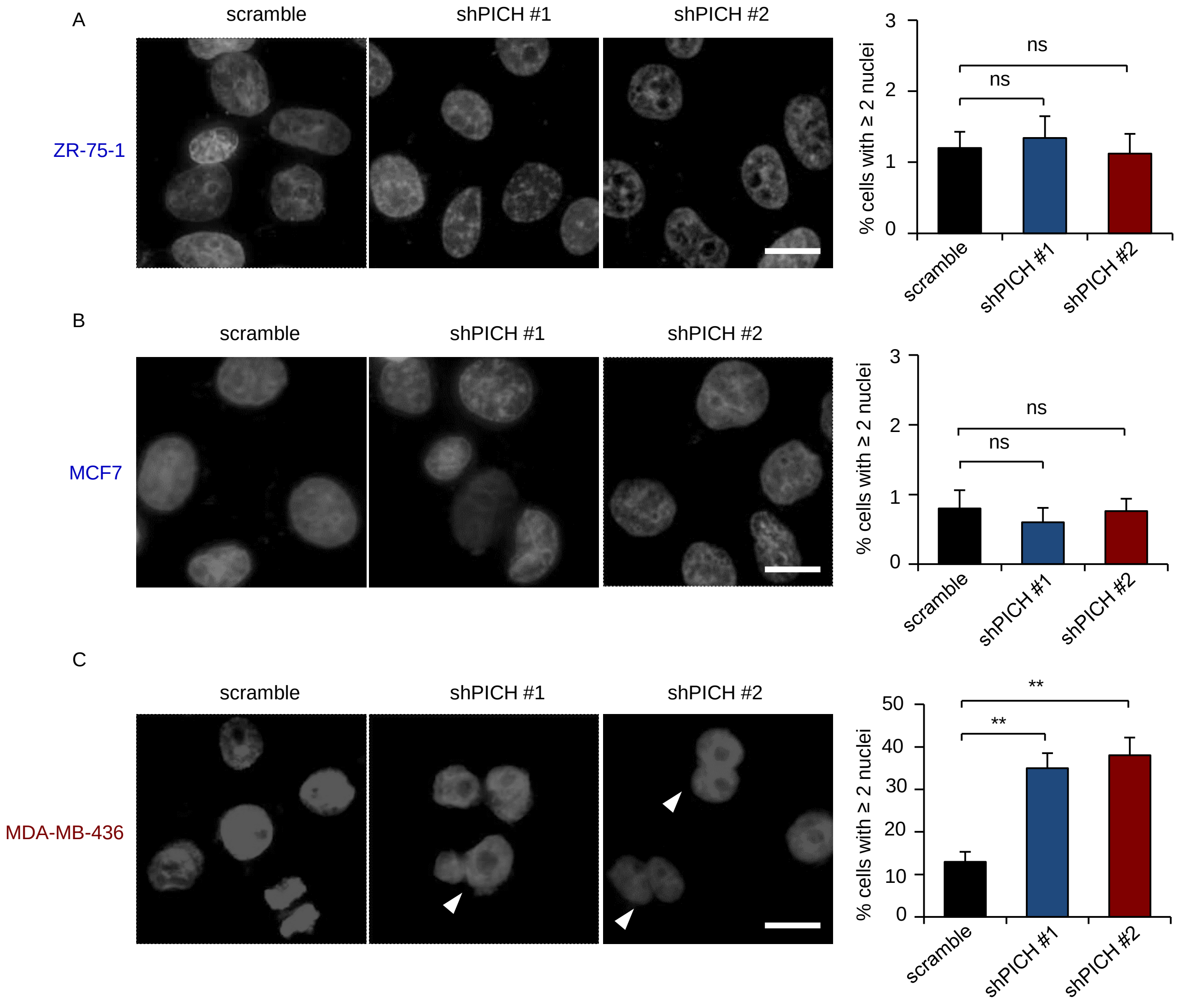

Figure S4. *PICH* depletion increases the percentage of binucleated cells in TNBC cells

(A-B) Luminal breast cancer cells (ZR-75-1, MCF7) were silenced for *PICH*. Control and *PICH*-silenced cells were stained with DAPI. Scale bar, 15 μm. (C) MDA-MB-436 cells overexpressing control shRNA or shPICH were cultured for three days and stained with DAPI. Scale bar, 15 μm. Binucleated cells was indicated by the white arrows in the representative images. (A-C) The percentage of binucleated cells was caculated. The bar graphs indicate means ± SD for three experiments. ***P* < 0.01; ns, not significant. Student's t-test.

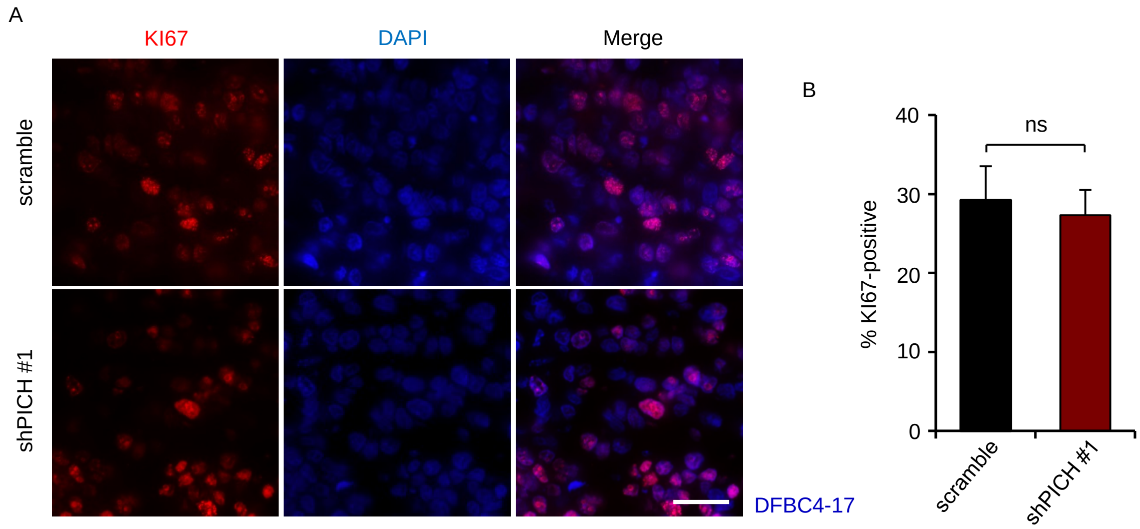

Figure S5. *PICH* knockdown does not inhibit primary luminal breast cancer cell growth *in vivo*

(A-B) The harvested DFBC4-17 tumors were fixed and subjected to KI67 staining. Representative micrographs (C) and quantification (D) of Ki67 staining (n = 3 mice in each treatment group) in tumors. Scale bar, 25 μm. Error bars represent means ± SEM. ns, not significant. Student's t-test.

Supplementary table 1. The list of PICH shRNA sequence and primers used for plasmid construction.

Name	Sequences
PLKO_scramble_F	CCGGGTGGACTCTTGAAAGTACTATCTCGAGATAGTACTTT CAAGACTCCACTTTTTG
PLKO_scramble_R	AATTAAAAAGTGGACTCTTGAAAGTACTATCTCGAGATAGTA CTTTCAAGAGTCCAC
PLKO_shPICH #1_F	CCGGACAAGATCTCTCCAGTATAAACTCGAGTTTATACTGG AGAGATCTTGTTTTTTG
PLKO_shPICH #1_R	AATTCAAAAAACAAGATCTCTCCAGTATAAACTCGAGTTTAT ACTGGAGAGATCTTGT
PLKO_shPICH #2_F	CCGGCCTGAAGATTATCCAGAAGAACTCGAGTTCTTCTGGA TAATCTTCAGGTTTTTG
PLKO_shPICH #2_R	AATTCAAAAACCTGAAGATTATCCAGAAGAACTCGAGTTCTT CTGGATAATCTTCAGG
GFP-PICH-res1-F	GAGGGTGAGAAACAAGACCTGTCAAGCATAAAGGTGAA
GFP-PICH-res1-R	GCTTGACAGGTCTTGTTTCTCACCCTCTTTGGGCAGATC
GFP-PICH-K128A-F	TGATATGGGATTAGGGGCGACTGTTCAA
GFP-PICH-K128A-R	GCCCCTAATCCCATATCATCAGCCAATAT
GFP-PICH-E229Q-F	ACTATGTCATCCTCGATCAAGCACATAAA
GFP-PICH-E229Q-R	GATCGAGGATGACATAGTCCCACACAAAC

Supplementary table 2. Pearson’s correlations of PICH expression with other cell cycle genes.

GENE 1	GENE 2	Corr.Coeff in TCGA	Corr.Coeff in METABRIC
PICH	KIF4A	0.864336	0.75065
PICH	CEP55	0.816545	0.69654
PICH	MKI67	0.808103	0.573626
PICH	MELK	0.816803	0.705607
PICH	BUB1	0.840622	0.714599
PICH	CENPA	0.764368	0.683573
PICH	AURKB	0.69435	0.672258
